# Worldwide Use of RUCAM for Causality Assessment in 81,856 Idiosyncratic DILI and 14,029 HILI Cases Published 1993–Mid 2020: A Comprehensive Analysis

**DOI:** 10.3390/medicines7100062

**Published:** 2020-09-29

**Authors:** Rolf Teschke, Gaby Danan

**Affiliations:** 1 Department of Internal Medicine II, Division of Gastroenterology and Hepatology, Klinikum Hanau, D-63450 Hanau, Teaching Hospital of the Medical Faculty of the Goethe University, D-60590 Frankfurt/Main, Germany; 2 Pharmacovigilance Consultancy, F-75020 Paris, France; gaby.danan@gmail.com

**Keywords:** RUCAM, Roussel Uclaf Causality Assessment Method, diagnostic algorithm, iDILI, iDrug induced liver injury, DILI, HILI, herb induced liver injury

## Abstract

**Background:** A large number of idiosyncratic drug induced liver injury (iDILI) and herb induced liver injury(HILI) cases of variable quality has been published but some are a matter of concern if the cases were not evaluated for causality using a robust causality assessment method (CAM) such as RUCAM (Roussel Uclaf Causality Assessment Method) as diagnostiinjuryc algorithm. The purpose of this analysis was to evaluate the worldwide use of RUCAM in iDILI and HILI cases. **Methods:** The PubMed database (1993–30 June 2020) was searched for articles by using the following key terms: Roussel Uclaf Causality Assessment Method; RUCAM; Idiosyncratic drug induced liver injury; iDILI; Herb induced liver injury; HILI. **Results:** Considering reports published worldwide since 1993, our analysis showed the use of RUCAM for causality assessment in 95,885 cases of liver injury including 81,856 cases of idiosyncratic DILI and 14,029 cases of HILI. Among the top countries providing RUCAM based DILI cases were, in decreasing order, China, the US, Germany, Korea, and Italy, with China, Korea, Germany, India, and the US as the top countries for HILI. **Conclusions:** Since 1993 RUCAM is certainly the most widely used method to assess causality in IDILI and HILI. This should encourage practitioner, experts, and regulatory agencies to use it in order to reinforce their diagnosis and to take sound decisions.

## 1. Introduction

Idiosyncratic drug induced liver injury (DILI), in short also termed iDILI, and herb induced liver injury (HILI) are complex diseases and received much attention in recent years [1,2,3,4,5,6,7,8,9]. The present scientometric study comprehensively analyzed the global knowledge base and specific emerging topics of DILI derived from 1995 publications in 79 countries and regions, with an impressive annual growth of reports between 2010 and 2019 and almost 340 studies published in 2020 [1]. In parallel, more and more publications on DILI and HILI cases refer to RUCAM (Roussel Uclaf Causality Assessment Method) for causality assessment [10,11,12,13]. The original RUCAM was first published in 1993 [14] and updated in 2016 [15] with additional information on its use and perspectives [16,17], which is now the preferred version to be used in future cases of DILI and HILI [15]. It is widely recognized that causality assessment in DILI and HILI is a multifaceted approach [7,8,9,15], a real medical challenge, for which a diagnostic quantitative algorithm such as RUCAM is an easy tool for case evaluation [10,11,12,13,14,15,16,17,18] to solve complex conditions [18].

The RUCAM algorithm is a structured, standardized, transparent, liver specific and quantitative diagnostic clinical scale based on key elements of liver injury, which are individually scored and provide a score for five-degree causality grading from unrelated up to highly probable causality levels [15]. Since key elements are specifically described and scored, assessments are objective with little risk of subjectivity [15,16,17] commonly observed if the approach to assess causality lacks scored key elements [19]. RUCAM can help expand our knowledge by enlarging population analysis with prospective and scored causality assessment, allowing for harmonized interpretation of data across populations [20]. In this context, RUCAM should be viewed as a cornerstone approach assessing causality of liver injury cases [15,16,17,21], because robust diagnostic biomarkers are rarely available due to misconducted studies as outlined by EMA (European Medicines Agency: Formerly London, UK, now Amsterdam, Netherlands) [21].

In this review article, current conditions of DILI and HILI cases assessed worldwide using RUCAM were critically analyzed. For the first time, the focus is on reports published from1993 to mid 2020 and the discussion of their potential use to describe specific features of DILI and HILI cases.

## 2. Literature Search and Source

The PubMed database (1993–30 June 2020) was searched for articles by using the following key terms: Roussel Uclaf Causality Assessment Method; RUCAM; Idiosyncratic drug induced liver injury (iDILI); Herb induced liver injury (HILI). Key terms were used alone or in combination. Limited to the English language, publications from each search terms were analyzed for suitability of this review article. The electronic search was completed on 30 June 2020 and supplemented by a manual literature search, using also the large private archive of the authors when the publication was not yet referenced in PubMed. The final compilation consisted of original papers including individual case reports and case series, consensus reports, and review articles with the most relevant publications included in the reference list of this review.

## 3. Definitions

RUCAM is presented as an algorithm that requires a few criteria allowing for a final quantitative evaluation. In particular, establishing RUCAM based criteria of liver test thresholds and liver injury patterns was revolutionary at the time of first publication issued from an international consensus meeting of experts, without the requirement of a liver biopsy [14] with same principles preserved in the updated RUCAM [15].

### 3.1. RUCAM Based Liver Injury

#### 3.1.1. Liver Test Thresholds

A liver injury caused by exogenous compounds such as drugs and herbs is defined by specific threshold values established for the liver tests (LTs) alanine aminotransferase (ALT) and alkaline phosphatase (ALP), with current serum activities considered as relevant for ALT ≥ 5 × ULN (upper limit of normal) and ALP ≥ 2 × ULN [15] provided that ALP is of hepatic origin. The original RUCAM was the first causality assessment method (CAM) ever considering threshold criteria although initially with lower values for ALT [15] as compared to currently used criteria [16]. Of note, serum bilirubin is not part of the diagnostic RUCAM algorithm that uses ALT or ALP as diagnostic liver test. In this context, conjugated bilirubin is a sign of the severity of the liver injury.

#### 3.1.2. Liver Injury Pattern

RUCAM was also the first CAM proposing different patterns of liver injury based on LTs [14] and are included also in the updated RUCAM [15]. To determine the liver injury pattern, the ratio R is to be calculated using the multiple of the ULN of serum ALT divided by the multiple of the ULN of serum ALP, provided the ALP increase is of hepatic origin. For causality assessment purposes, two types of liver injury are defined (independently from histological findings): first a hepatocellular injury with R > 5, and second, a cholestatic/mixed liver injury with R ≤ 5.

### 3.2. Idiosyncratic Versus Intrinsic Liver Injury

Liver injury is either idiosyncratic, due to the interaction between the exogenous synthetic chemical or phytochemical and a susceptible individual with some genetic factor(s), or it is intrinsic due to chemical overdose [11,12,13]. In the present analysis, idiosyncratic injury is considered, as opposed to intrinsic liver injury most commonly observed with overdosed drugs such as acetaminophen [22].

## 4. Worldwide Publications of DILI

The current scientometric report from China on knowledge mapping confirmed the high worldwide interest in DILI publications and identified a total of 1995 DILI studies published between 2010 and 2019, although information on the applied method of causality assessment was not provided and will need further clarification [1]. This Chinese analysis on the top 10 countries involved in DILI research listed the US, China, Japan, Germany, UK, Spain, France, the Netherlands, Sweden, and Canada. In addition, many interesting details on DILI were comprehensively discussed with focus on definition, incidence rate, clinical characteristics, etiology or pathogenesis such as the character of the innate immune system, the regulation of cell-death pathways, susceptible HLA (Human Leukocyte Antigen) identification, or criteria and methods of causality assessment, all topics were considered as the knowledge base for DILI research [1].

## 5. Worldwide Publications of RUCAM Based Idiosyncratic DILI

The worldwide impact of DILI can best be quantified by using liver injury cases assessed for causality with a robust method that allows for establishing causality gradings for each implicated drug and to exclude alternative causes unrelated to drug administration.

### 5.1. Countries and Regions

In the current analysis, authors from 31 countries worldwide reported on cases of idiosyncratic DILI caused by multiple drugs published from 1993 up to mid 2020 and applied in all cases RUCAM to assess causality (Table 1) [23,24,25,26,27,28,29,30,31,32,33,34,35,36,37,38,39,40,41,42,43,44,45,46,47,48,49,50,51,52,53,54,55,56,57,58,59,60,61,62,63,64,65,66,67,68,69,70,71,72,73,74,75,76,77,78,79,80,81,82,83,84,85,86,87,88,89,90,91,92,93,94,95,96,97,98,99,100,101,102,103,104,105,106,107,108,109,110,111,112,113,114,115,116,117,118,119,120,121,122,123,124,125,126,127,128,129,130,131,132,133,134,135,136,137,138,139,140,141,142,143,144,145,146,147,148,149,150,151,152,153,154,155,156,157,158,159,160,161,162,163,164,165,166,167,168,169,170,171,172,173,174,175,176,177,178,179,180]. Such a table with a comprehensive list of publications over a long period has never been reported before and will facilitate the search for RUCAM based DILI cases caused by individual drugs, considering that databases such as LiverTox may have problems providing real DILI cases [10,74].

### 5.2. Hospital and Other Sources

RUCAM based DILI cases were mostly published by authors from university hospitals and their affiliated teaching hospitals known for their high reputation (Table 1). Among these were a broad range of departments, which in most cases include departments of Hepatology and Gastroenterology, ensuring careful clinical evaluation of patients with suspected DILI and associated causality assessment for the offending drug(s). To a lesser degree, other departments were contributors, for instance, Pharmacology, or Pharmacy and Pharmaceutical sciences [170].

In addition to hospitals, other sources provided RUCAM based DILI cases (Table 1). Among these were National Institutes of Health from Japan [92] and the US [165], consortia from Spain [115,141], the adverse drug reactions advisory committee (ADRAC) from Sweden [126], regulatory pharmacovigilance and pharmacoepidemiology centers from France [58,59] and Italy [86], drug commission of medical association from Germany [64], committee for drug induced liver injury from China [42]; also, drug reaction reporting database from Spain [65], regulatory agency from Spain [114] health insurance from the US [157], and drug safety departments of drug companies from France [57], Sweden {148], and Switzerland [132]. Some of these played an eminent role in promoting the use of RUCAM in prospective studies, particularly those from Spain [115], Sweden [126], and the US with France and Sweden [148].

### 5.3. Top Ranking Countries

Among the top 10 countries were in decreasing order China, the US, Germany, Korea, Italy, Sweden, Spain, Japan, Argentina, and Thailand, whereby the top 5 countries provided most of the DILI cases (Table 2). Authors from these 5 countries contributed together 75,133 DILI cases out of a total 81,856 worldwide DILI cases, corresponding to 91.8%. On the lower part of the list ranked the 6 countries Israel, Malaysia, Mexico, Morocco, Saudi Arabia, and Turkey, authors from these low ranking countries provided each one single DILI case assessed for causality using RUCAM, corresponding to 6 cases altogether out of a total of 81,856 DILI cases. Authors from the remaining 20 countries with a ranking from 6 down to 25 contributed 6.723 DILI cases out of overall 81,856 cases corresponding to 8.2%. In essence, RUCAM based DILI cases were mostly published in English language journals, raising the question how DILI cases were assessed and published by the other countries in local journals in languages other than English. Currently, overall 81,856 cases of idiosyncratic DILI assessed for causality by RUCAM have been retrieved via PubMed, all published 1993–June 2020 (Table 1) [23,24,25,26,27,28,29,30,31,32,33,34,35,36,37,38,39,40,41,42,43,44,45,46,47,48,49,50,51,52,53,54,55,56,57,58,59,60,61,62,63,64,65,66,67,68,69,70,71,72,73,74,75,76,77,78,79,80,81,82,83,84,85,86,87,88,89,90,91,92,93,94,95,96,97,98,99,100,101,102,103,104,105,106,107,108,109,110,111,112,113,114,115,116,117,118,119,120,121,122,123,124,125,126,127,128,129,130,131,132,133,134,135,136,137,138,139,140,141,142,143,144,145,146,147,148,149,150,151,152,153,154,155,156,157,158,159,160,161,162,163,164,165,166,167,168,169,170,171,172,173,174,175,176,177,178,179,180].

### 5.4. Annual Growth Trends of RUCAM Based DILI Case Publications

Analyses of growth trends provided additional information after identification of a total 1995 DILI studies, published between 2010 and 2019 but not stratified for causality assessment using RUCAM [1]. In the frame of the present analysis, only publications of idiosyncratic DILI cases were included if they had been assessed for causality using RUCAM, providing a more homogenous series with established DILI diagnoses.

#### 5.4.1. Published Annual RUCAM Based DILI Cases

**Figure 1:**
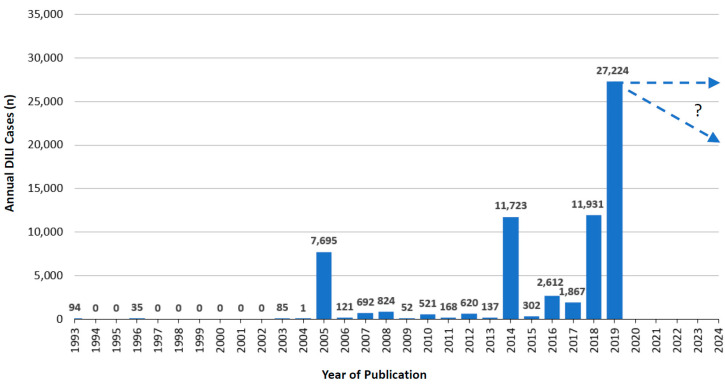
Annual cases of DILI assessed for causality by RUCAM and published since 1993.

Considering the period from 1993 to 2019, annually published cases of RUCAM based idiosyncratic DILI ranged between 0 and 27,224 in 2019, but data of 2020 were not included because case counting stopped by end of June in this particular year (Figure 1). Three phases of trends appeared with respect to published RUCAM based DILI cases: (1) phase 1 with clinical field testing from 1996 to 2004 (2) phase 2 with promotion from 2005 to 2013, and (3) phase 3 of worldwide use from 2014 to 2019.

Phase 1 started after the launch of RUCAM in 1993 [16,47] and the analysis of 94 DILI cases [47], the number of subsequent annual published DILI cases remained small until 2004, reaching 121 cases (Figure 1). This was the period of initial testing the RUCAM algorithm under clinical field conditions with interesting early information provided by 3 reports [58,91,114]. The first report came from Spain, was published in 1996, analyzed a major study cohort of DILI due to amoxicillin and clavulanate, and described their typical clinical features, with Rodríguez as first author and Zimmerman as senior author [114] who actually was involved as an expert from the US in the international consensus meetings [14] but did not promote RUCAM in DILI evaluations in his own country. Of interest was also the retrospective design of this analysis, suggesting that this particular study approach is feasible [114] although a prospective approach is recommended [15]. The second report was from Japan with Japanese patients, published in 2003 by Masumoto et al. [91]. This study favored RUCAM over other CAMs, provided evidence that the performance of the lymphocyte transformation test was poor in line with previous reports, and the RUCAM criteria were viewed as useful for diagnosing DILI in Japanese patients. The third publication came from France, reported in 2004 on details of a patient with DILI by pioglitazone, and showed the feasibility of a good case report to be assessed by RUCAM, evaluated by Arotcarena et al. [58]. All three reports were hallmarks of the first phase of RUCAM based DILI case series devoted to clinical field evaluation that ended in 2004 (Figure 1).

Phase 2 started in 2005 with overall 7695 annually published RUCAM based DILI cases (Figure 1) [115,126,147,148]. Among these were 461 cases provided by Andrade et al. from Spain retrieved from a prospective study involving various drugs [115], additional 784 cases from Sweden were published by Björnsson and Olsson retrieved from a prospective study of DILI by various drugs [126], whereas from the US 2 case reports of DILI by amoxicillin and clavulanate were presented by Fontana et al. [147] as well as a large cohort of DILI caused by ximelagatran occurred in clinical trials was published by Lee et al. [148]. These 4 studies promoted the usefulness of RUCAM evaluating DILI cases [115,126,147,148] by preferring a prospective study design [115,126], evaluating single DILI case reports [147], and correctly assessing suspected DILI cases in clinical trials [148]. Whereas RUCAM had already a firm place among DILI experts in Europe, it seems that experts in the US became more familiar with the use and practicability of RUCAM.

Phase 3 is characterized by the worldwide use of RUCAM for DILI started in 2014 with 11,525 DILI cases (Figure 1), mostly attributed to one study with 11,109 DILI cases provided by Cheetham et al. [157]. Starting in 2015, there was a continuous rise of published RUCAM based DILI cases (Figure 1), likely driven also by the updated RUCAM available online 2015 and published in 2016 [15]. With 27,224 published DILI cases, the maximum level on an annual base was achieved in 2019 (Figure 1). Until end of June 2020, additional 15,153 published DILI cases were counted but not included in Figure 1, corresponding already to more than half of the cases counted in 2019 and representing a good base for 2020 and further years.

#### 5.4.2. Annual RUCAM Based DILI Publications and Growth Trend

**Figure 2:**
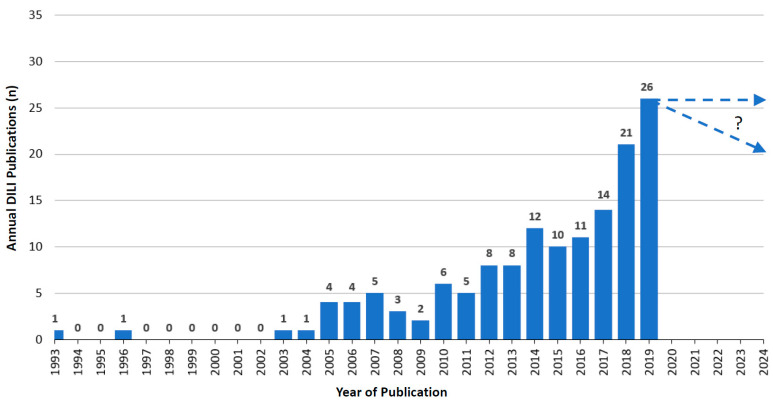
Annual publications of DILI cases assessed for causality by RUCAM as reported since 1993.

Over the years starting from 1993, when RUCAM was launched [14,57], and until 2019 an upward trend of annual RUCAM based DILI publications can be observed with some dips in between (Figure 2). In 2019, 26 publications were counted, and 15 publications from January 2020 until end of June 2020 that were not included in the listing (Figure 2). Overall 158 publications with RUCAM based DILI cases were counted from 1993 until mid 2020 (Table 1).

### 5.5. Specificities of DILI Case Evaluation

Large study cohorts of RUCAM based DILI cases accumulated many different drugs and provided as expected a global information of the DILI cases due to various drugs without a detailed description of clinical features drug by drug (Table 1). Consequently, typical clinical features of a DILI by a single drug cannot be obtained from large cohorts as opposed to single DILI case reports or case series that included DILI cases due to a single drug (Table 1). In general, studies with a single DILI case or a few cases are more informative because they provide an exhaustive past medical history with clinical details required for a sound case evaluation. In search for typical DILI features by specific drugs, therefore, assistance may be provided by the drug listing (Table 1). In addition, details can be retrieved via the internet, using the search terms drug induced liver injury and the name of the suspected drug, combined with RUCAM or the updated RUCAM.

### 5.6. Worldwide Top Ranking of Drugs Causing DILI

There is concern how best to establish a top ranking of drugs most commonly implicated in DILI [70,74]. A recent study presented a list with top ranking drugs out of overall 3312 DILI cases evaluated by RUCAM (Table 3) [70]. The RUCAM based DILI cases were retrievd from 15 reports by six national databases of DILI registries and three large medical centers worldwide, which provided the DILI cases under consideration. Contributing countries and regions were in alphabetical order China, Germany, Latin America, Iceland, India, Singapore, Spain, Sweden, and the US. It was found that the databases of national registries and large medical centers are the best sources of drugs implicated in DILI cases. There is also the note that presently DILI cases of the LiverTox database are less suitable for clinical or regulatory purposes as presented on its website because many suspected DILI cases were derived from published cases of poor quality, lacking a robust CAM such as RUCAM [70,74]. Consequently, the majority of LiverTox based cases of assumed DILI could previously not be classified as real DILI [74]. To overcome these diagnostic shortcomings, LiverTox attempted a top ranking of drugs by counting the published DILI cases for each individual drug [74]. It was assumed that the degree of causality probability increases with the number of published DILI reports: the higher the case number the higher the probability. This special approach explains the variability of the top listing presented by liverTox [74] as compared to RUCAM based cohorts [70].

## 6. Worldwide Publications of HILI Cases Assessed for Causality Using RUCAM

Highlights of liver injury cases have been reported not only for DILI but with increasing frequency also for HILI cases questionable due to lack of a robust CAM [7,8,9]. The problems associated with HILI are specifically addressed in the current analysis, which considers for the first time worldwide HILI cases using RUCAM as a robust algorithm for assessing causality.

### 6.1. Countries and Regions

Authors from many countries around the world reported on cases of HILI in connection with the consumption of various herbs, all published since 1993 (Table 4) [29,37,38,42,48,100,101,102,103,113,115,116,117,118,181,182,183,184,185,186,187,188,189,190,191,192,193,194,195,196,197,198,199,200,201,202,203,204,205,206,207,208,209,210,211,212,213,214,215,216,217,218,219,220,221,222,223,224,225,226,227,228,229,230,231,232,233,234,235,236,237,238,239,240,241,242,243,244,245,246,247,248,249,250,251,252,253,254,255]. Specifically considered were patients, who experienced HILI with established causality using RUCAM. Such a table with a comprehensive list of publications over a long period of time will help the search for RUCAM based HILI cases caused by specific herbs or herbal products containing a mixture of several herbs. This list is unique as compared to databases that may have problems providing real HILI cases not confounded by alternative causes or lack of a robust causality assessment.

### 6.2. Hospital and Other Sources

Most RUCAM based HILI cases were provided by authors from university hospitals and their affiliated teaching hospitals with their departments of Hepatology and Gastroenterology, Medicine or Internal Medicine (Table 3). Rare contributors were other departments like those with focus on Emergency Medicine [255], Clinical Pharmacology and Toxicology in Berlin [209], Pharmacology and Toxicology in Hannover [213], Pharmacy in Singapore [238], Physiology and Pharmacology in Rome [219,221], Anatomical, Histological, Forensic and Orthopedic Sciences in Rome [222], Pediatrics in Seoul [234], and among the contributors were even the Neurology and Headache Center in Essen [212] and Spine and Joint Research Institute in Seoul [235].

Other sources providing RUCAM based HILI cases include the Chinese Academy of Medical Sciences in Beijing [195], School of Chinese Materia Medica in Beijing [199,202], Competence Centre for Complementary Medicine and Naturopathy in Munich [211], Biomedical Research and Innovation Platform South African Medical Research Council in Tygerberg [239], United States Pharmacopeia in Rockville [254], and Center of Pharmacovigilance of Florence [218].

### 6.3. Top Ranking Countries

Among the countries presenting RUCAM based HILI cases were on top in descending order China and Korea, followed by Germany, India and the US, whereby the top 5 countries provided most of the HILI cases (Table 5). Authors from these 5 countries contributed together 13,808 HILI cases out of a total 14,029 worldwide HILI cases, corresponding to 98.4%. On the lower part of the list ranked the 4 countries Brazil, Colombia, Switzerland, and Turkey, authors from these low ranking countries provided each one HILI case assessed for causality using RUCAM, corresponding to 4 cases altogether out of a total of 14,029 HILI cases. Authors from the remaining 20 countries with a ranking from 6 down to 14 contributed 217 HILI cases out of overall 14,029 cases corresponding to almost 1.6%.

#### 6.3.1. Published Annual RUCAM Based HILI Cases

**Figure 3:**
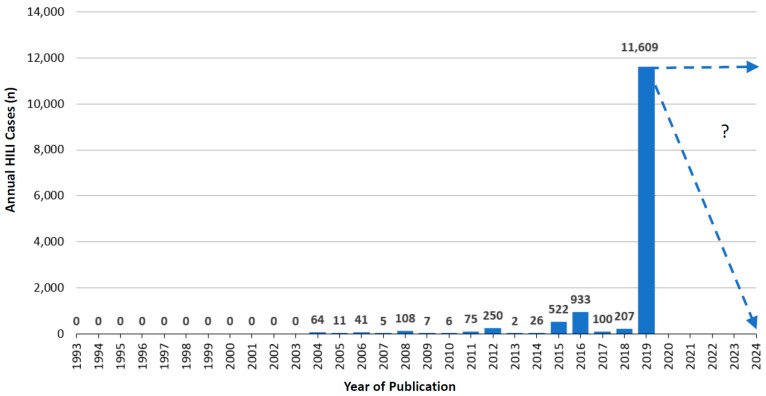
Annual cases of HILI cases assessed for causality by RUCAM and published since 1993.

From 1993 to 2019, published annual cases of RUCAM based HILI ranged between 0 and 11,609 in 2019, while 57 HILI cases of 2020 were not included because case counting stopped by end of June in this particular year (Figure 3). Three phases of trends appeared with respect to published RUCAM based HILI cases: (1) phase 1 with lack of any clinical field testing from 1993 to 2003, (2) phase 2 with slow promotion from 2004 to 2016, and (3) phase 3 of worldwide use from 2017 to 2019.

Phase 1 started after the launch of RUCAM in 1993 [16,47] but without a single published HILI case until 2003 (Figure 3). The lack of published RUCAM based HILI cases during this period might be due to the fact that the value of RUCAM was not yet sufficiently known or to uncertainties whether herbs have the potential to cause liver injury. In addition, the term of herb induced liver injury or its acronym HILI was unknown at that time and therefore not in common use.

During the subsequent phase 2, the number of annual published HILI cases remained small with cases ranging from 2 to 933, considering the years from 2004 until 2016 (Figure 3). In 2008, there were 108 HILI cases, with 18 Spanish cases published by García-Cortés et al. [117,241] and 90 Korean cases published by Kang et al. [227] and Sohn et al. [228]. During 2016, there was a sharp increase with 933 HILI cases, mostly attributed to 866 cases from China published by Zhu et al. [42]. As a reminder and outlined recently, herb induced liver injury with HILI as its acronym was first introduced and proposed as a specific term in the scientific literature only in 2011 [12]. This may explain retarded publications on HILI cases (Figure 3).

Phase 3 started with low HILI case numbers in 2017 and 2018 (Figure 3), considering that the updated RUCAM applicable also to HILI cases was published only in 2016 [15]. With 11,609 the largest HILI case number was published in 2019 (Figure 3) as a consequence of the ongoing worldwide use of RUCAM for assessing causality in suspected HILI cases (Table 4). In particular, contributing countries were in alphabetical order Australia [29], Brazil [183], China [48,196,197,198,199,200,201], Germany [213,214,215], India [217], Italy [222], Korea [103], Spain [115,117,240,241,242], Switzerland [244], and the US [245,246,247,248,249,250,251,252,253,254]. Most of the 11,619 HILI cases published in 2019 were from China [48,196] and Korea [103], with 6971 cases published by Shen et al. [48], 2019 cases reported by Byeon et al. [103], and 1552 cases provided by Chow et al. [196]. However, until mid 2020 only 57 HILI cases were published (Table 4) [202,203,253,254], suggesting for the whole year 2020 at best 100 cases (Figure 3).

#### 6.3.2. Annual RUCAM Based HILI Publications and Growth Trend

**Figure 4:**
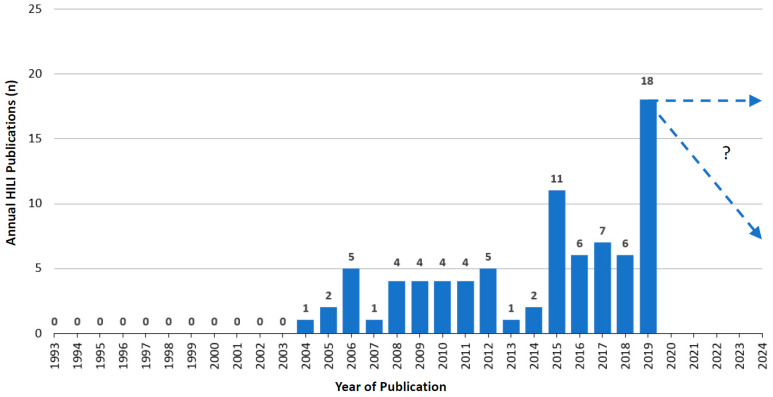
Annual publications of HILI cases assessed for causality by RUCAM as reported since 1993.

Over the years starting from 1993, when RUCAM was launched [14,57] and until 2019, an upward trend of annual RUCAM based HILI publications can be observed with some dips in between (Figure 4). In 2019, 18 publications were counted and 4 publications until end of June 2020 that were not included (Figure 4). For the whole year 2020, therefore, at best perhaps 8 publications can be anticipated (Figure 4). These figures show that a total of 85 publications with RUCAM based HILI cases were reported from 1993 until mid 2020 (Table 2).

### 6.4. Specificities of HILI Cases

Large study cohorts of RUCAM based HILI cases accumulate many different herbs and provide as expected a global information of many HILI cases without a detailed description of clinical features for specific herbs (Table 4). Consequently, studies with a single or a few HILI cases have many advantages because they focus on a single herb or herbal product causing the liver injury and usually provide an exhaustive past medical history with clinical details required for a sound case evaluation. For interested physicians, regulators, and manufacturers, this listing provides individual cases with herbs causing HILI.

## 7. Utility of RUCAM

The utility of RUCAM has been confirmed in in many liver injury cases of DILI (Table 1) and HILI (Table 4) published from countries and regions around the world, as outlined in various reports [5,11,15,16,17,18] and briefly summarized (Table 6). In short, the high qualification of RUCAM as an objective diagnostic algoritm to assess causality in liver injury cases of DILI and HILI is the clue of its increasing use (Figure 1, Figure 2, Figure 3 and Figure 4). RUCAM is smoothly applied by clinicians or regulators and obviously without problems (Table 1 and Table 4). The worldwide use allows data comparison among different countries, a unique condition for multifacetted diseases as DILI and HILI are. RUCAM is also applied in epidemiology studies. Finally and most importantly, each individual DILI and HILI case report contain important details of liver injury cases that may be helpful for physicians in care of patients with suspected DILI and HILI.

## 8. Other CAMs

Apart from the objective diagnostic RUCAM algorithm, a few non-RUCAM based CAMs are known, critically discussed elsewhere in detail [5,15]. In short, they are less accurate than RUCAM, not quantitative as not based on specific elements to be scored individually, not specific for liver injury cases, not structured, not validated, or based on individual arbitrary subjective opinions. In fact, other CAMs are still caught up in the pre-RUCAM and pre-AI era [18] and thereby neglecting the use of diagnostic algorithms such as the original RUCAM [14] or the now preferred updated version [18].

## 9. Limitation of the Analysis

The current analysis is based on published data of DILI and HILI reports in English, or at least an abstract in English, rather than on unpublished data contained in the original data sets that were not available to the authors of the analysis for re-analysis. Although most of the published DILI and HILI cases provide excellent data, some authors forgot presenting RUCAM based causality gradings or included cases with a possible causality grading in their final evaluations of cases together with a probable or highly probable causality level. Nevertheless, a broad range of different causality gradings was commonly provided in most published cases, respective references allow for detailed information. As being outside the scope of this article, causality gradings for individual reports were not provided (Table 1 and Table 5), but some details of 46,266 DILI cases assessed by RUCAM were published earlier [11]. Problematic are study cohorts with inclusion of both DILI and HILI cases, unless both groups were separately evaluated [48]. As expected, not all of the patients were commonly confirmed as being DILI by RUCAM scoring, but the number of published cases remained accurate. For instance, special conditions are evident in the randomized clinical trial of ximelagatran [148]. In this prospective, report, hepatic findings were analyzed in all suspected cases with regard to causal relationship to ximelagatran by using RUCAM, considered as the most reliable tool to assess causality [148]. Applying RUCAM based on ALT thresholds only is insufficient since 92% of the ximelagatran group did not meet this criterion missing then a final robust causality grading, as opposed to 8% of the study group receiving partially high causality gradings. This study reaffirms the utility of RUCAM to identify cases with real DILI cases in cohorts under real world conditions.

## 10. Outlook

The perspectives using the updated RUCAM in future DILI and HILI cases are favorable because many authors including those from the US become more familiar with RUCAM and are ready to use this diagnostic algorithm (Table 1, Table 2, Table 3 and Table 4), in line with principles of Artificial Intelligence to solve difficult processes [18]. Moreover, as in the US and many other countries RUCAM was successfully used to assess causality in cases of DILI, there is no need to invent another instrument specifically designed for drug development [255]. The issue of overlooked alternative causes remains a clinical problem and was described already in 1999 by Aithal et al. [256] and guided by RUCAM subsequently confirmed [69,257].

Future DILI and HILI studies should adhere on a prospective study design as strongly recommended in the RUCAM updated in 2016 because a retrospective approach may create concern on the validity of the published results due to incomplete information [15]. Neglecting this recommendation and using instead a retrospective design could be problematic [48]. In addition, attempts to lift RUCAM based causality gradings from possible to probable must be resisted [48]. Discouraged is in particular the use of a non-RUCAM based CAM in addition to RUCAM, because such a combination causes uncertainty due to disputable results of causality gradings. It is not recommended to mix in the same cohort patients with DILI or HILI [48] because this situation will complicate a separate evaluation of DILI or HILI features. However, it is clear that in individual cases RUCAM allows for a distinction between a drug and a medicinal herb when causality gradings are different.

## 11. Conclusions

The current analysis showed a favorable run of the RUCAM algorithm globally used since its launch in 1993, considering the annually published DILI and HILI cases. Overall 95,885 liver injury cases were published using RUCAM for causality assessment, namely 81,856 iDILI cases and 14,029 HILI cases. The global use of RUCAM assessing causality in cases of DILI and HILI helps compare study results among various countries and facilitates description of typical clinical features, best derived from case reports or small case series. RUCAM solves complex conditions as an algorithm in line with principles of Artificial Intelligence. Top ranking countries providing RUCAM based DILI cases were China, the United States, Germany, Korea, and Italy, whereas most RUCAM based HILI cases were published by authors from China, Korea, Germany, India, and the United States. In term of number of cases published, there is no other causality assessment method that could outperform RUCAM evaluating DILI and HILI cases. This should encourage all the stakeholders involved in DILI and HILI to systematically use RUCAM in order to reinforce their diagnosis and take the right decisions for the benefit of the patients.

## Figures and Tables

**Table 1:** Worldwide countries with a selection of published DILI cases assessed for causality using RUCAM.

Country/ DILI Cases, *n*	First Author/Year	DILI Cases, *n*	Drugs	Comments on RUCAM Based DILI Cases
**Argentina** *n* = 625	Bessone, 2016 [23]	197	Various drugs	DILI caused by a variety of drugs, not allowing individual description of features
	Bessone, 2019 [24]	114	Various drugs	Individual drugs not available for DILI feature characterization
	Colaci, 2019 [25]	311	Various drugs	DILI features for single drugs were not presented
	García, 2019 [26]	3	Methotrexate	Feature details provided for DILI by methotrexate
**Australia** *n* = 106	Lin, 2014 [27]	47	Various volatile anaesthetics	DILI by anesthetics without individual features of isoflurane, desflurane, or sevoflurane
	Ahmed, 2015 [28]	1	Ipilimumab	Detailed features of the DILI case
	Laube, 2019 [29]	1	Atorvastatin	Good feature presentation of this DILI case
	Worland, 2020 [30]	57	Infliximab	Feature presentation of DILI by the drug
**Bahrain** *n* = 25	Sridharan, 2020 [31]	25	Various antiepileptic drugs	No feature details provided of DILI due to individual drugs
**Brazil** *n* = 4	Becker, 2019 [32]	4	Various drugs	Features of DILI caused by some drugs
**Canada** *n* = 4	Yan, 2006 [33]	2	Rofecoxib	Two well described case features of DILI caused by rofecoxib
	Nhean, 2019 [34]	2	Dolutegravir	Careful described features of DILI
**China** *n* = 35,825	Hou, 2012 [35]	300	Various drugs	No feature details available for DILI by individual drugs
	Lv, 2012 [36]	89	Various drugs	Specific features of DILI by individual drugs were not presented
	Hao, 2014 [37]	140	Anti-Tuberculotics	Lacking specific DILI features of any drug
	Ou, 2015 [38]	231	Various drugs	No feature specifics of DILI are available for individual drugs
	Zhu, 2015 [39]	39	Various drugs	Specific features of DILI caused by individual drugs were not provided
	Lu, 2016 [40]	513	Various drugs	Missing specific features of DILI caused by individual drugs
	Yang, 2016 [41]	124	Various drugs	Feature specifics of DILI caused by individual drugs were not provided
	Zhu, 2016 [42]	870	Various drugs	No specific features of DILI by individual drugs were presented
	Naqiong, 2017 [43]	157	Various statins	Cohort consisted of patients with DILI caused by atorvastatin, simvastatin, and rosuvastatin, but specific features were not provided for individual statins
	Li, 2018 [44]	1	Iguratmod	Detailed feature description of DILI
	Song, 2018 [45]	1	Posaconazole	Careful feature presentation of DILI by this drug
	Tao, 2018, [46]	290	Anti-Tuberculotics	Cohort included patients with DILI caused by isoniazid, rifampin, pyrazinamide, ethambutol, and streptomycin, but specific features were not presented for individual drugs
	Liao, 2019 [47]	1	Cefepime	Well described features of DILI by this drug
	Shen, 2019 [48]	18,956	Various drugs	Cohort comprized patients with DILI, but special DILI features related to individual drugs were not published.
	Xing, 2019 [49]	133	Various drugs	No specific feature presentation of DILI by individual drugs
	Ma, 2020 [50]	1	Fenofibrate	Specific feature of DILI by this drug presented
	Tao, 2020 [51]	146	Anti-Tuberculotics	Lacking feature data of DILI caused by individual drugs
	Wang, 2020 [52]	155	Anti-Tuberculotics	Cohort included patients with DILI due to not further identified anti-TB regimens, hence attributing specific DILI features to individual drugs was not possible
	Yang, 2020 [53]	13,678	Various drugs	No feature details of DILI by individual drugs
**Colombia** *n* = 19	Ríos, 2013 [54]	1	Albendazole	Detailed feature description of DILI caused by albendazole
	Cano-Paniagua, 2019 [55]	18	Various drugs	Perfect feature description of DILI by drugs in this excellent prospective epidemiology study using the updated RUCAM for causality assessment
**Egypt** *n* = 75	Alhaddad, 2020 [56]	75	Various drugs	Feature details of DILI by individual drugs incompletely provided
**France** *n* = 170	Bénichou, 1993 [57]	94	Various drugs	No detailed feature description of DILI by the drugs
	Arotcarena, 2004 [58]	1	Pioglitazone	Feature description of the case
	Moch, 2012 [59]	18	Etifoxine	Detailed features of DILI due to etifoxine treatment
	Carrier, 2013 [60]	1	Methyl-prednisolone	Features of DILI well described for the drug
	Ripault, 2013 [61]	1	Crizotinib	Good feature details provided for DILI by this drug
	Dumortier, 2017 [62]	5	Methyl-prednisolone	Careful feature description of DILI caused by the drug
	Meunier, 2018 [63]	50	Nimesulide	No feature description of DILI by this drug
**Germany** *n* = 10,907	Stammschulte, 2012 [64]	37	Flupirtine	Carefully presented features of DILI caused by flupirtine
	Douros, 2014 [65]	7	Flupirtine	Comprehensive feature presentation of DILI due to flupirtine
	Douros, 2014 [66]	198	Various drugs	Cohort of patients with DILI associated with the use of various drugs, but special features of DILI by individual drugs were not provided
	Buechter, 2018 [67]	15	Various drugs	No detailed feature presentation of DILI caused by individual drugs
	Dragoi, 2018 [68]	16	Diclofenac	Cohort of DILI patients with presentation of limited specific DILI features
	Teschke, 2018 [69]	7278	Various drugs	Cohort of DILI patients without feature specification for individual drugs
	Teschke, 2018 [70]	3312	Various drugs	Cohort of DILI cases not providing special features of DILI by individual drugs
	Weber, 2019 [71]	44	Various drugs	No specific features of DILI caused by individual drugs provided
**Iceland** *n* = 367	Björnsson, 2012 [72]	73	Statins	Specific feature details provided of DILI by statins
	Björnsson, 2013 [73]	72	Various drugs	Cohort of DILI cases without providing typical features of DILI by single drugs
	Björnsson, 2016 [74]	222	Various drugs	The two assessed cohorts provided no typical features of DILI caused by the evaluated drugs
**India** *n* = 424	Harugeri, 2009 [75]	1	Montelukast	Good feature presentation of a patient with DILI caused by montelukast
	Devarbhavi, 2010 [76]	313	Various drugs	No feature description of DILI due to individual drugs
	Rathi, 2017 [77]	82	Various drugs	Cohort of DILI cases but features of DILI by indivual drugs were not presented
	Taneja, 2017 [78]	2	Etodolac	Detailed feature presentation of DILI
	Das, 2018 [79]	24	Various drugs	Cohort with limited feature description of few patients with DILI caused by drugs assessed for causality by RUCAM or other CAMs
	Dutta, 2020 [80]	1	Haloperidol	Perfect presented feature details of DILI caused by this drug
	Kulkarni, 2020 [81]	1	Vitamin A	Perfect feature details of this DILI case
**Israel** *n* = 1	Gluck, 2011 [82]	1	Amiodarone	Careful feature description of a patient with DILI caused by a single drug
**Italy** *n* = 1562	Rigato, 2007 [83]	1	Flavoxate	Good feature presentation of a patient with DILI caused by flavoxate
	Licata, 2010 [84]	46	Various drugs including Nimesulide	Feature description of patients with DILI by nimesulide but no description for DILI by other drugs
	Abenavoli, 2013 [85]	1	Cyproterone acetate	Detailed feature description of a patient with DILI
	Ferrajolo, 2017 [86]	938	Various antibiotics	Combined feature presentation of all antibiotics causing DILI in paediatric patients
	Licata, 2017 [87]	185	Various drugs	Epidemiology study, hence no feature description of patients with DILI by any drug
	Giacomelli, 2018 [88]	362	Nevirapine	Detailed feature description of DILI by nevirapine observed in all patients
	Licata, 2018 [89]	28	Rivaroxaban	Perfect feature description of this DILI cohort
	Lovero, 2018 [90]	1	Ustekinumab	Careful feature description of DILI caused by this drug
**Japan** *n* = 939	Masumoto, 2003 [91]	85	Various drugs	No detailed feature description of DILI caused by drugs
	Hanatani, 2014 [92]	182	Various drugs	Detailed features of DILI by individual drugs not provided
	Niijima, 2017 [93]	1	Ipragliflozin	Provided case features of DILI
	Ji, 2017 [94]	1	Methimazole	Perfect feature presentation of DILI caused by this drug
	Aiso, 2019 [95]	270	Various drugs	Global feature description of DILI by all drugs
	Kishimoto, 2019 [96]	1	Clonazepam	Detailed feature of DILI by this drug
	Hiraki, 2019 [97]	1	Tegafur-Uracil	Good feature presentation of DILI caused by the drug
	Kakisaki, 2019 [98]	398	Various drugs	Perfect feature description of the cohort
**Korea** *n* = 6528	Choi, 2008 [99]	1	Albendazole	Detailed feature description of DILI by albendazole in a case report of a single patient
	Suk, 2012 [100]	101	Various drugs	Lacking detailed feature description of DILI by individual drugs
	Son, 2015 [101]	1	Various drugs	No specific feature description of DILI due to comedication
	Woo, 2016 [102]	1	Various comedicated drugs	Lacking specific feature description of DILI due to comedication
	Byeon, 2019 [103]	6391	Various drugs	Missing specific feature of DILI caused by individual drugs
	Kwon, 2019 [104]	33	Nimesulide	Detailed feature description of DILI caused by nimesulide, using a prospective study design in this perfect analysis
**Malaysia** *n* = 1	Thalha, 2018 [105]	1	Kombiglyze	Perfect feature description of DILI caused by the combination of metformin and saxagliptin
**Mexico** *n* = 1	Lammel-Lindemann, 2018 [106]	1	Candesartan	Well described features of DILI by this drug
**Morocco** *n* = 1	Essaid, 2010 [107]	1	Tadalafil	Feature description of DILI due to the drug
**Pakistan** *n* = 264	Abid, 2020 [108]	264	Various drugs	No specific feature details presented for DILI caused by individual drugs
**Portugal** *n* = 53	Costa-Moreira, 2020 [109]	53	Various drugs	Specific feature details of DILI caused by individual drugs were not provided
**Saudi Arabia** *n* = 1	Alqrinawi, 2020 [110]	1	Menotropin	Perfect feature details of DILI by this specific drug
**Serbia** *n* = 99	Miljkovic, 2010 [111]	80	Various drugs	No detailed feature description of DILI by individual drugs
	Miljkovic, 2011 [112]	19	Various drugs	Lacking detailed feature presentation of DILI caused by individual drugs
**Singapore** *n* = 14	Wai, 2006 [113]	14	Various drugs	Limited feature description of DILI
**Spain** *n* = 1181	Rodríguez, 1996 [114]	35	Various drugs	No feature details of DILI cases provided for individual drugs
	Andrade, 2005 [115]	461	Various drugs	Limited feature description of DILI case details
	Andrade, 2006 [116]	28	Various drugs	Partial feature description of DILI by few drugs
	García-Cortés, 2008 [117]	225	Various drugs	Limited feature description of DILI by few drug groups
	Lucena, 2011 [118]	78	Amoxicillin Clavulanate	Careful feature description of DILI by the drug combination
	Lucena, 2011 [119]	9	Various drugs	Feature description of DILI caused by individual drugs
	Robles-Diaz, 2015 [120]	25	Anabolic and androgenetic steroids	Limited feature description of DILI
	Tong, 2015 [121]	1	Methylphenidate	Careful evaluation of feature details provided for this DILI case
	Medina-Calitz, 2016 [122]	298	Various drugs	No specific feature description for DILI by any drug
	López-Riera, 2018 [123]	17	Various drugs	Lack of specific feature presentation of DILI by any drug
	Machlab, 2019 [124]	1	Apixabam	Careful feature description of the DILI case caused by the drug
	Zoubek, 2019 [125]	3	Methyl-prednisolone	Perfect feature presentation of DILI
**Sweden** *n* = 1508	Björnsson, 2005 [126]	784	Various drugs	Detailed feature description of DILI by few drugs
	De Valle, 2006 [127]	77	Various drugs	Limited feature description of DILI by a few drugs
	Björnsson, 2007 [128]	77	Various drugs	Lacking substantial feature description of DILI caused by few drugs
	Björnsson, 2007 [129]	570	Various drugs	Feature details of DILI presented
**Switzerland** *n* = 68	Goossens, 2013 [130]	1	Ibandronate	Detailed description of immune DILI features by ibandronate, a biphosphonate
	Russmann, 2014 [131]	14	Rivaroxaban	Perfect individual feature presentation of each DILI case
	Scalfaro, 2017 [132]	49	Sacubitril Valsartan	No feature details of DILI caused by the drugs
	Schneider, 2017 [133]	1	Zoledronic acid	Feature details provided for DILI by this drug
	Terziroli Beretta-Piccoli, 2018 [134]	1	Atovaquon/Proguanil	Detailed feature description of DILI
	Visentin, 2018 [135]	2	NSAID Amoxicillin/Clavulanate	Lack of feature details of DILI by individual drugs
**Thailand** *n* = 509	Treeprasertsuk, 2010 [136]	80	Various antibiotics	No feature details provided for DILI caused by individual drugs in the context of this epidemiology study
	Sobhonslidsuk, 2016 [137]	383	Various drugs	Missing feature details of DILI caused by individual drugs
	Chayanupatkul, 2020 [138]	46	Various drugs	Feature details of DILI due to individual drugs not provided
	Duzenli, 2019 [139]	1	Phenprobamate	Detailed feature description of DILI by this drug
	Hussaini, 2007 [140]	43	Various antibiotics	No feature details of DILI caused by individual drugs
	Daly, 2009 [141]	51	Flucloxacillin	Excellent feature details presented for DILI cases
**Turkey** *n* = 1	Spraggs, 2011 [142]	61	Laptinib	Excellent feature description of DILI case
**United** **Kingdom** *n* = 263	Islam, 2014 [143]	1	Anastrazole	Feature presentation of a patient with DILI due to anastrazole
	Dyson, 2016 [144]	1	Sofosbuvir	Features of DILI by this drug provided
	Abbara, 2017 [145]	105	Various anti-Tuberculotics	Feature presentation of all DILI cases due to various anti-tuberculosis drugs
	Vliegenthart, 2017 [146]	1	Nitrofurantoin	Feature description of DILI by the drug
**United States** *n* = 20,311	Fontana, 2005 [147]	2	Amoxicillin, Amoxicillin/Clavulanate	Well described features of DILI caused by the drugs
	Lee, 2005 [148]	6448	Ximelagatran	Perfect presentation of DILI features
	Stojanovski, 2007 [149]	1	Atomoxetine	Good DILI case feature presentation
	Lammert, 2008 [150]	598	Various drugs	No feature presentation of DILI by individual drugs
	Singla, 2010 [151]	1	Cephalexin	DILI features of a single case
	Nabha, 2012 [152]	1	Etravirine	Presentation of DILI feature
	Sprague, 2012 [153]	1	Varenicline	Careful DILI feature description
	Markova, 2013 [154]	56	Bosentan	Some global feature description
	Marumoto, 2013 [155]	4	NSAID	Limited feature details of DILI cases
	Bohm, 2014 [156]	1	Daptomycin	DILI feature description in this case
	Cheetham, 2014 [157]	11,109	Various drugs	No specific feature presentation of any drug under consideration
	Lim, 2014 [158]	1	Various drugs	No presentation of specific features of DILI by 4 drugs used concomitantly or sequentially
	Russo, 2014 [159]	22	Statins	No individual feature description for DILI caused by various statins
	Veluswamy, 2014 [160]	1	Polamidomide	Feature presentation of DILI
	Baig, 2015 [161]	1	Rivaroxaban	Good feature decription of this DILI case
	Hammerstrom, 2015 [162]	1	Amblodipine	Feature presentation of DILI caused by this drug
	Stine, 2015 [163]	2	Simeprevir	Detailed feature presentation of the DILI cases
	Tang, 2015 [164]	1	Bupropion, doxycycline	Complex feature presentation of DILI due to comedication
	Unger, 2016 [165]	1	Ciprofloxacin	Detailed feature description of DILI by this single drug
	Gharia, 2017 [166]	1	Letrozole	Perfect feature presentation of DILI
	Nicoletti, 2017 [167]	339	Various drugs	No specific feature details of DILI caused by individual drugs
	Gayam, 2018 [168]	3	Various drugs	Feature details of DILI by the drugs
	Hayashi, 2018 [169]	493	Various drugs	Lacking specific feature details of DILI by individual drugs
	Patel, 2018 [170]	1	Everolimus	Specific features described for DILI by this drug
	Shamberg, 2018 [171]	34	Various drugs	No specific features of DILI caused by individual drugs presented
	Cirulli, 2019 [172]	268	Various drugs	Feature details of DILI by individual drugs not provided
	Nicoletti, 2019 [173]	197	Flucloxacillin	Specific feature details of DILI by flucloxacillin were not provided
	Sandritter, 2019 [174]	1	Various drugs	No feature description of DILI by individual drugs
	Shumar, 2019 [175]	1	Memantine	Detailed feature description of DILI by this drug
	Tsung, 2019 [176]	70	Pembrolizumab	Features described in detail for DILI caused by this drug
	Xie, 2019 [177]	1	Anastrozole	Feature details of DILI presented
	Ghabril, 2020 [178]	551	Various drugs	No feature details of DILI by individual drugs
	Mullins, 2020 [179]	99	Micafungin	Feature details presented of DILI by this drug

Abbreviations: CAMs, Causality Assessment Methods; DILI, Drug induced liver injury; NSAID, Non-steroidal anti-inflammatory drug; RUCAM, Roussel Uclaf Causality Assessment Method.

**Table 2:** Top ranking of countries providing DILI cases assessed for causality by RUCAM.

Top Ranking Countries	Cases, *n*	References
**1. China**	35,825	[35,36,37,38,39,40,41,42,43,44,45,46,47,48,49,50,51,52,53]
**2. United States**	20,311	[147,148,149,150,151,152,153,154,155,156,157,158,159,160,161,162,163,164,165,166,167,168,169,170,171,172,173,174,175,176,177,178,179]
**3. Germany**	10,907	[64,65,66,67,68,69,70,71]
**4. Korea**	6528	[99,100,101,102,103,104]
**5. Italy**	1562	[83,84,85,86,87,88,89,90]
**6. Sweden**	1508	[126,127,128,129]
**7. Spain**	1181	[114,115,116,117,118,119,120,121,122,123,124,125]
**8. Japan**	939	[91,92,93,94,95,96,97,98]
**9. Argentina**	625	[23,24,25,26]
**10.Thailand**	509	[136,137,138]
**11. India**	424	[75,76,77,78,79,80,81]
**12. Iceland**	367	[72,73,74]
**13. Pakistan**	264	[108]
**14. UK**	263	[140,141,142,143,144,145,146]
**15. France**	170	[57,58,59,60,61,62,63]
**16. Australia**	106	[27,28,29,30]
**17. Serbia**	99	[111,112]
**18. Egypt**	75	[56]
**19. Switzerland**	68	[130,131,132,133,134,135]
**20. Portugal**	53	[109]
**21. Bahrain**	25	[31]
**22. Colombia**	19	[54]
**23. Singapore**	14	[113]
**24. Brazil**	4	[32]
**25. Canada**	4	[33,34]
**26. Israel**	1	[82]
**27. Malaysia**	1	[105]
**28. Mexico**	1	[106]
**29. Morocco**	1	[107]
**30. Saudi Arabia**	1	[110]
**31. Turkey**	1	[139]

Abbreviations: DILI, Drug induced liver injury; RUCAM, Roussel Uclaf Causality Assessment Method.

**Table 3:** Worlwide top ranking of drugs causing DILI cases with causality assessment by RUCAM.

Drug	RUCAM Based DILI Cases (*n*)
**1. Amoxicillin-clavulanate**	333
**2. Flucloxacilllin**	130
**3. Atorvastatin**	50
**4. Disulfiram**	48
**5. Diclofenac**	46
**6. Simvastatin**	41
**7. Carbamazepine**	38
**8. Ibuprofen**	37
**9. Erythromycin**	27
**10. Anabolic steroids**	26
**11. Phenytoin**	22
**12. Sulfamethoxazole/Trimethoprim**	21
**13. Isoniazid**	19
**14. Ticlopidine**	19
**15. Azathioprine/6-Mercaptopurine**	17
**16. Contraceptives**	17
**17. Flutamide**	17
**18. Halothane**	15
**19. Nimesulide**	13
**20. Valproate**	13
**21. Chlorpromazine**	11
**22. Nitrofurantoin**	11
**23. Methotrexate**	8
**24. Rifampicin**	7
**25. Sulfazalazine**	7
**26. Pyrazinamide**	6
**27. Gold salts**	5
**28. Sulindac**	5
**29. Amiodarone**	4
**30. Interferon beta**	3
**31. Propylthiouracil**	2
**32. Allopurinol**	1
**33. Hydralazine**	1
**34. Infliximab**	1
**35. Interferon alpha/Peginterferon**	1
**36. Ketaconazole**	1
**37. Busulfan**	0
**38. Dantrolene**	0
**39. Didanosine**	0
**40. Efavirenz**	0
**41. Floxuridine**	0
**42. Methyldopa**	0
**43. Minocycline**	0
**44. Telithromycin**	0
**45. Nevirapine**	0
**46. Quinidine**	0
**47. Sulfonamides**	0
**48. Thioguanine**	0

Substantially modified from a previous report [70], which provides references for each implicated drug.

**Table 4:** Worldwide countries with a selection of published HILI cases assessed for causality using RUCAM.

Country/ HILI Cases, *n*	First Author/ Year	HILI Cases, *n*	Herbal Products	Comments on RUCAM Based HILI Cases
**Australia** *n* = 2	Smith, 2016 [181]	1	*Garcinia Cambogia*	Careful feature detail description of HILI by this herb
	Laube, 2019 [29]	1	Ginseng	Feature presentation of this single HILI case
**Austria** *n* = 2	Stadlbauer, 2005 [182]	2	Various herbs contained in Tahitian NONI juice	Features described for these HILI cases
**Brazil** *n* = 1	Barcelos, 2019 [183]	1	*Senecio brasiliensis*	Complete feature description of HSOS caused by this herb
**China** *n* = 10,914	Yuen, 2006 [184]	7	Various herbs	No specific feature description of HILI by individual herbs
	Cheung, 2009 [185]	3	*Psoralea corylifolia*	Well described features of HILI cases
	Chau, 2011 [186]	27	Various herbs	Lacking feature presentation of HILI by individual herbs
	Lin, 2011 [187]	1	*Gynura segetum*	Perfect feature presentation of HSOS
	Gao, 2012 [188]	5	*Gynura segetum*	Excellent feature description of HSOS
	Lai, 2012 [189]	74	Various herbs *Polygonum multiflorum*	Missing feature presentation of HILI by individual herbs
	Dong, 2014 [190]	18	Various herbs	Good feature presentation of HILI
	Hao, 2014 [37]	8	PA containing herbs	Lacking feature presentation of HILI by individual herbs
	Gao, 2015 [191]	23	Various herbs	Perfect feature presentation of HSOS cases
	Ou, 2015 [38]	130	*Polygonum multiflorum*	No feature description of HILI caused by the herb
	Wang, 2015 [192]	40	*Polygonum multiflorum*	Comprehensive feature description of HILI cases
	Zhu, 2015 [193]	158	*Polygonum multiflorum*	Detailed feature presentation of HILI cases
	Zhang, 2016 [194]	54	Various herbs	No feature details described of HILI by individual herbs
	Zhu, 2016 [42]	866	Various herbs	Missing feature details of HILI caused by individual herbs
	Li, 2017 [195]	1	*Polygonum multiflorum*	Excellent description of feature details provided for HILI case
	Chow, 2019 [196]	1552	Various herbs	No feature presentation of HILI by individual herbs but excellent listings
	Jing, 2019 [197]	145	*Polygonum multiflorum*	Missing feature presentation of HILI caused by individual herbs
	Li, 2019 [198]	1	*Psoralea corylifolia*	Perfect feature presentation of HILI
	Ni, 2019 [199]	331	*Polygonum multiflorum*	Feature description of HILI cases
	Shen, 2019 [48]	6971	Various herbs	No feature details presented for HILI by individual herbs
	Tan, 2019 [200]	3	*Swietenia macrophylla*	Perfect presentation of feature details for these HILI cases
	Zhu, 2019 [201]	488	Various herbs	Lacking feature details of HILI caused by individual herbs
	Gao, 2020 [202]	1	*Psoralea*	Feature description of this HILI case
	Xia, 2020 [203]	7	*Swietenia macrophylla*, syn skyfruit	Careful description of HILI features
**Colombia** *n* = 1	Cárdenas, 2006 [204]	1	*Polygomun multiflorum*	Features well described for this HILI case
**France** *n* = 10	Parlati, 2017 [205]	10	*Aloe vera*	Excellent feature presentation of the HILI cases
**Germany** *n* = 170	Teschke, 2009 [206]	1	Ayurveda herbs	Complete feature presentation of the HILI case
	Teschke, 2011 [207]	22	*Chelidonium majus* syn. Greater Celandine	Complete feature description provided for HILI cases
	Teschke, 2012 [208]	21	*Chelidonium majus* syn. Greater Celandine	Thorough features presented of HILI cases
	Douros, 2015 [209]	10	Various herbs	No detailed features reported of HILI caused by individual herbs
	Teschke, 2015 [210]	12	*Camellia sinensis*, syn. Green tea, or Lu Cha	Feature details presented of HILI cases
	Melchart, 2017 [211]	26	Herbal TCMs	Well described features of HILI caused by individual TCM herbs
	Diener, 2018 [212]	10	*Petasites hybridus*	Provided features of HILI by this herb
	Anderson, 2019 [213]	48	*Petasites hybridus*	Description of HILI features
	Gerhardt, 2019 [214]	1	*Chelidonium majus*, syn. Greater Celandine	HILI features described
	Teschke, 2019 [215]	19	*Camellia sinensis*	Careful feature presentation of HILI cases
**India** *n* = 117	Philips, 2018 [216]	94	Ayurvedic and other herbs	Features not individually described for HILI by various herbs
	Philips, 2019 [217]	17	Various herbs	No detailed features presented for HILI cases by individual herbs
**Italy** *n* = 77	Lapi, 2010 [218]	1	*Serenoa repens*	Detailed feature presentation of HILI
	Mazzanti, 2015 [219]	19	*Camellia sinensis*, syn. green tea	Careful feature description of HILI cases
	Sáez-González, 2016 [220]	1	*Chelidonium majus*	Feature details of the HILI case
	Mazzanti, 2017 [221]	55	*Red yeast rice*	Thorough feature presentation of HILI
	Osborne, 2019 [222]	1	*Mitragyna speciosa*, syn. Kraton	Individual feature details not provided for the HILI case
**Japan** *n* = 3	Tsuda, 2010 [223]	1	Saireito	Perfect feature details presented for the HILI case
	Hisamochi, 2013 [224]	2	*Agaricus blazei Murill*	Excellent presentation of HILI features
**Korea** *n* = 2507	Ahn, 2004 [225]	64	Various herbs	Missing feature presentation of HILI by individual herbs
	Seo, 2006 [226]	17	Various herbs	No individual feature description of HILI by specific herbs
	Kang, 2008 [227]	66	Various herbs	Lacking feature details of HILI by individual herbs
	Sohn, 2008 [228]	24	Various herbs	Feature details of HILI by individual herbs not provided
	Kang, 2009 [229]	1	*Corydalis speciosa*	Perfect feature details provided for this single HILI
	Kim, 2009 [230]	2	Arrowroot, syn. ge Gen	Excellent presentation of features for these HILI cases
	Bae, 2010 [231]	1	*Polygonum multiflorum*	Careful feature details presented for this HILI case
	Yang, 2010 [232]	3	*Aloe vera* or *arborescens*	Thorough description of features of these HILI cases
	Jung, 2011 [233]	25	*Polygonum*	Excellent feature presentation of the HILI cases
	Kim, 2012 [234]	1	*multiflorum*	Perfect feature description for this HILI case
	Suk, 2012 [100]	149	*Hovenia dulcis*, syn. Juguju	No feature description of HILI caused by individual herbs
	Lee, 2015 [235]	27	Various herbs	Lacking feature details of HILI caused by individual herbs
	Lee, 2015 [236]	97	Various herbs	Feature details of HILI cases caused by individual herbs were not provided
	Woo, 2015 [102]	5	Various herbs	No feature details presented for HILI by individual herbs
	Cho, 2017 [237]	6	Various herbs	Missing feature details of HILI cases by individual herbs
	Byeon, 2019 [103]	2019	Various herbs	No detailed feature description of HILI by individual herbs
**Singapore** *n* = 25	Wai, 2006 [113]	15	Various herbs	No detailed features presented for HILI cases by individual herbs
	Teo, 2016 [238]	10	Various herbs	Missing feature details of HILI cases
**South Africa** *n* = 47	Awortwe, 2018 [239]	47	Various herbs	Features were not provided for cases of HILI caused by individual herbs
**Spain** *n* = 46	Andrade, 2005 [115]	9	Various herbs	No feature details of HILI by individual herbs
	Jimenez-Saenz, 2006 [240]	1	*Camellia sinensis*	Feature details presented for this HILI case
	García-Cortés, 2008 [241]	13	Various herbs	Lacking feature details of HILI caused by individual herbs
	García-Cortés, 2008 [117]	5	Various herbs	Specific feature details of HILI by individual herbs not provided
	Medina-Caliz, 2018 [242]	18	*Camellia sinensis* and other herbs	No specific feature details provided for HILI
**Sweden** *n* = 5	Björnsson, 2007 [243]	5	*Camellia sinensis*	Feature details provided for HILI cases
**Switzerland** *n* = 1	Ruperti-Repilado, 2019 [244]	1	*Artemisia annua*	Careful feature presentation of this HILI case
**Turkey** *n* = 1	Yilmaz, 2015 [245]	1	Lesser Celandine, syn. Pilewort	Excellent feature presentation of the HILI case
**United States** *n* = 100	Papafragkakis, 2016 [246]	1	Chinese skullcap plus Black catechu	Perfect feature presentation of this HILI case
	Dalal, 2017 [247]	1	Ayurvedic herb	Specific case features described
	Kesavarapu, 2017 [248]	1	Yogi Detox tea with multiple herbs	Individual specific features not provided for this HILI case caused specifically by a single herb
	Kothadia, 2018 [249]	19	*Garcinia Cambogia*	Careful feature presentation of HILI cases
	Surapaneni, 2018 [250]	19	*Camellia sinensis*	Feature details provided for the HILI case
	Imam, 2019 [251]	1	Curcumin	Thorough feature description of the HILI case
	Yousaf, 2019 [252]	9	*Garcinia Cambogia*	Excellent feature description of the HILI case
	Oketch-Rabah, 2020 [253]	29	*Camellia sinensis* extract	Perfect feature presentation of HILI by this herb
	Schimmel, 2020 [254]	20	*Mitragyna speciosa*, syn. Kraton	Feature details provided for HILI cases

Abbreviations: DILI, Drug induced liver injury; HILI, Herb induced liver injury; HSOS, Hepatic sinusoidal obstruction syndrome; RUCAM, Roussel Uclaf Causality Assessment Method, TCM, Traditional Chinese Medicines; USP, United States Pharmacopeia; WHO, World Health Organizations.

**Table 5:** Top ranking of countries providing HILI cases assessed for causality by RUCAM.

Top Ranking Countries	Cases, *n*	References
**1. China**	10,914	[37,38,42,48,184,185,186,187,188,189,190,191,192,193,194,195,196,197,198,199,200,201,202,203]
**2. Korea**	2507	[100,101,102,103,225,226,227,228,229,230,231,232,233,234,235,236,237]
**3. Germany**	170	[206,207,208,209,210,211,212,213,214,215]
**4. India**	117	[216,217]
**5. US**	100	[247,248,249,250,251,252,253,254,255]
**6. Italy**	77	[218,219,220,221,222]
**7. South Africa**	47	[239]
**8. Spain**	46	[115,117,240,241,242]
**9. Singapore**	25	[113,238]
**10. France**	10	[205]
**11. Sweden**	5	[244]
**12. Japan**	3	[223,224]
**13. Australia**	2	[29,181]
**14. Austria**	2	[182]
**15. Brazil**	1	[183]
**16. Colombia**	1	[204]
**17. Switzerland**	1	[245]
**18. Turkey**	1	[246]

Abbreviations: HILI, Herb induced liver injury; RUCAM, Roussel Uclaf Causality Assessment Method.

**Table 6:** Characteristics of RUCAM.

RUCAM Specificities
**Basic features**
Validated method (gold standard) based on cases with positive reexposure test results, providing thereby a robust CAM
Worldwide use with 46,266 DILI cases assessed by RUCAM published 2014–2019, outperforming thereby any other CAM in term of number of cases published
Assesses causality in DILI and HILI cases validly and reproducibly
A typical intelligent diagnostic algorithm in line with artificial intelligence (AI) concepts
A diagnostic algorithm for objective, robust causality assessment
Assessment is user friendly, cost effective with results available in time and without needing expert rounds that often provide subjective and fragile, arbitrary opinions based on own experience
Transparency of case data and clear result presentation
Suitable for reevaluation by peers and any of other interested parties such as national regulatory agencies international registries, and pharma companies
Mandatory application for DILI cases if to be used for establishing new robust diagnostic biomarkers
High causality gradings with complete data
With prospective case data collection best results are obtainable
**Clearly defined and scored key elements**
Time frame of latency period
Time frame of dechallenge
Recurrent ALT or ALP increase
Risk factors
Individual comedications
Exclusion of alternative causes
Markers of HAV, HBV, HCV, HEV
Markers of CMV, EBV, HSV, VZV
Cardiac hepatopathy and other alternative causes
Liver and biliary tract imaging
Doppler sonography of liver vessels
Prior known hepatotoxicity of drug or herb
Unintentional reexposure
**Other important specificities**
Laboratory based hepatotoxicity criteria
Laboratory based liver injury pattern
Hepatotoxicity specific method
Structured, liver related method
Quantitative, liver related method, based on scored key elements

Abbreviations: AI: Artificial Intelligence; ALT: Alanine aminotransferase; ALP: Alkaline phosphatase; CAM: Causality assessment method; CMV: Cytomegalovirus; DILI: Drug induced liver injury; EBV: Epstein Barr virus; HAV: Hepatitis A virus; HBV: Hepatitis B virus; HCV: Hepatitis C virus; HEV: Hepatitis E virus; HILI: herb induced liver injury; HSV: Herpes simplex virus; RUCAM: Roussel Uclaf Causality Assessment Method; VZV: Varicella zoster virus.
